# The fate of murine double minute X (MdmX) is dictated by distinct signaling pathways through murine double minute 2 (Mdm2)

**DOI:** 10.18632/oncotarget.22320

**Published:** 2017-11-06

**Authors:** Paula M. Hauck, Eric R. Wolf, David J. Olivos, Ciaran P. McAtarsney, Lindsey D. Mayo

**Affiliations:** ^1^ Department of Pediatrics, Herman B Wells Center for Pediatrics Research, Indianapolis, Indiana, 46202, United States of America; ^2^ Department of Biochemistry and Molecular Biology, Indiana University School of Medicine, Indianapolis, Indiana, 46202, United States of America; ^3^ Indiana University Simon Cancer Center, Indiana University School of Medicine, Indianapolis, Indiana, 46202, United States of America

**Keywords:** MdmX, Mdm2, neddylation, MLN4924, ATM

## Abstract

Mouse double minute 2 (Mdm2) and MdmX dimerize in response to low levels of genotoxic stress to function in a ubiquitinating complex, which signals for destabilization of p53. Under growth conditions, Mdm2 functions as a neddylating ligase, but the importance and extent of MdmX involvement in this process are largely unknown. Here we show that when Mdm2 functions as a neddylating enzyme, MdmX is stabilized. Furthermore, we demonstrate that under growth conditions, MdmX enhances the neddylation activity of Mdm2 on p53 and is a substrate for neddylation itself. Importantly, MdmX knockdown in MCF-7 breast cancer cells resulted in diminished neddylated p53, suggesting that MdmX is important for Mdm2-mediated neddylation. Supporting this finding, the lack of MdmX in transient assays or in *p53/MdmX-/- MEFs* results in decreased or altered neddylation of p53 respectively; therefore, MdmX is a critical component of the Mdm2-mediated neddylating complex. c-Src is the upstream activator of this Mdm2-MdmX neddylating pathway and loss of Src signaling leads to the destabilization of MdmX that is dependent on the RING (Really Interesting New Gene) domain of MdmX. Treatment with a small molecule inhibitor of neddylation, MLN4924, results in the activation of Ataxia Telangiectasia Mutated (ATM). ATM phosphorylates Mdm2, converting Mdm2 to a ubiquitinating enzyme which leads to the destabilization of MdmX. These data show how distinct signaling pathways engage neddylating or ubiquitinating activities and impact the Mdm2-MdmX axis.

## INTRODUCTION

Post-translational modifications such as ubiquitin, sumo, nedd8, and ISG15, are essential for a myriad of fundamental processes in the cell. These modifications can induce conformational changes to recruit binding partners, prevent or enhance interactions, or result in changes in cellular localization. E1, E2 and E3 enzymes are responsible for conjugating these covalent modifications to proteins. Mouse double minute 2 (Mdm2) is one of over a thousand E3 ubiquitin ligases. Mdm2 is an oncoprotein that is elevated in 10% of all human cancers and 40-80% of high-grade human tumors [1, 2]. It is most widely recognized for its role in facilitating ubiquitination of p53, which can lead to p53 destabilization under conditions of genotoxic stress.

In response to low levels of DNA damage, Mdm2 has been described to act: as a monomer after phosphorylation by Ataxia Telangiectasia Mutated (ATM) [3, 4], as a homodimer [4–10], or as a heterodimer with MdmX [5-9, 11-15] after phosphorylation by c-Abl [16] in a ubiquitination complex. The post-translational modifications that dictate whether Mdm2 acts as a monomer, homo- or heterodimer are only beginning to be elucidated. While the Mdm2-MdmX heterodimer has been shown to have ubiquitin ligase activity [11], it is most commonly accepted that MdmX does not have ubiquitin ligase activity by itself. Most studies have investigated MdmX-Mdm2-p53 signaling under DNA damage, and agree that MdmX is destabilized under these conditions due to Mdm2-mediated ubiquitination of MdmX (reviewed by [17]). MdmX is related to Mdm2 (C-terminal domains are 46% identical), and can bind directly to the transactivation domain of p53 to inhibit p53 transcriptional activity [18]. MdmX is also considered an oncogene by its ability to transform primary cells and is amplified in approximately 65-85% of infiltrating ductal breast carcinomas [19], retinoblastomas [20] and metastatic melanomas [21].

Interestingly, Mdm2 is one of a small number of E3 ligases that can mediate neddylation [22]. We previously demonstrated that under growth conditions, Mdm2 is a substrate for Src phosphorylation at Y281 and Y302, which converts Mdm2 from a ubiquitin to a Nedd8 E3 ligase [23]. This Nedd8 activity of Mdm2 results in neddylated p53 that has increased stability but is transcriptionally inactive.

In the current study, we show that conversion of Mdm2 to a neddylating enzyme by growth signaling results in Mdm2 forming a heterodimer with MdmX. This dimerization is dependent on Src phosphorylation of Mdm2. We also show that MdmX is conjugated with Nedd8. As expected, in the presence of Src, Mdm2, and MdmX, p53 neddylation is enhanced, thereby blocking p53 transcriptional activity. However, inhibition of the E1 neddylating enzyme by MLN4924 (Pevonedistat -Takeda /Millennium Pharmaceuticals, Inc.) leads to the activation of p53. This is a multi-faceted mechanism whereby MdmX levels are dramatically decreased in addition to the prevention of neddylation. This destabilization is dependent on Mdm2. Our data show how multiple signaling pathways regulate the stability of MdmX and the regulation of p53.

## RESULTS

### Modulation of MdmX levels in response to post-translational modifications

**Figure 1:**
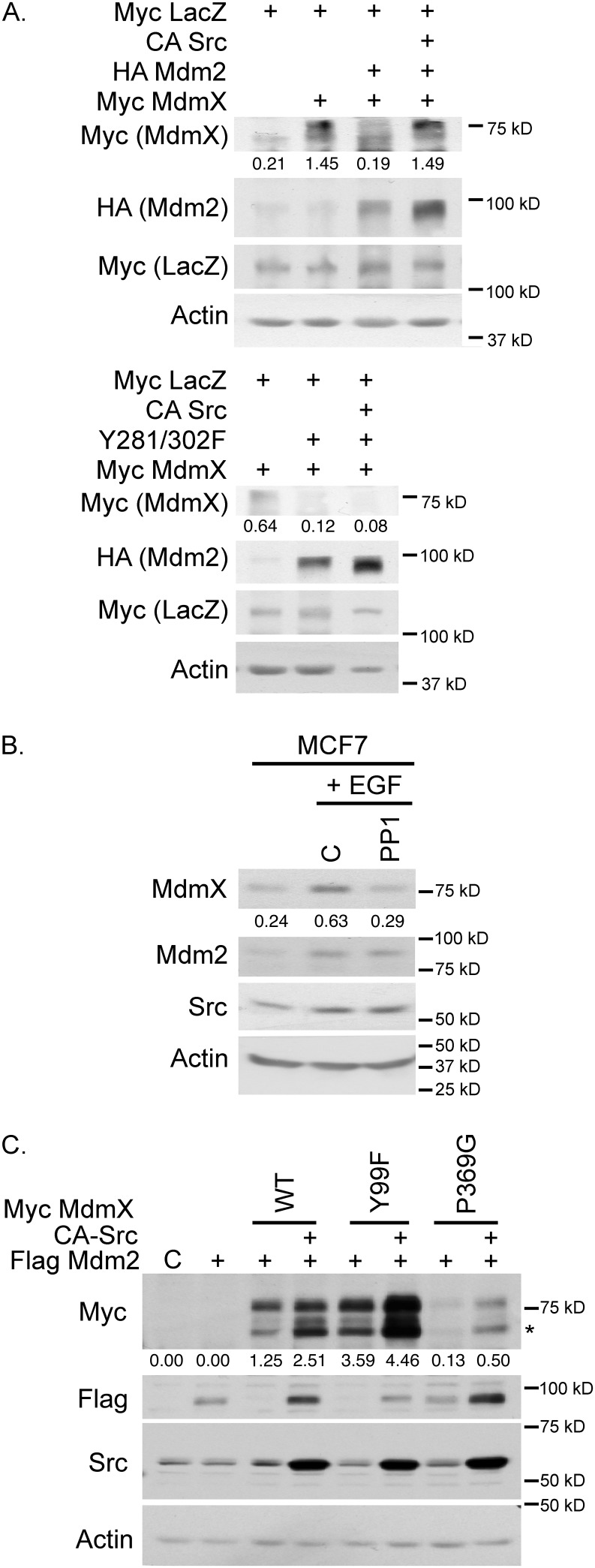
Conversion of Mdm2 to a neddylating ligase results in increased levels of MdmX as detected by western blot analysis **(A)** Without CA-Src in the H1299 transient transfections, Mdm2 acts as a ubiquitinating ligase and MdmX levels are decreased. The addition of CA-Src results in recovery of MdmX levels (top). The Y281-302F (Src phosphorylation sites) Mdm2 mutant cannot act as a neddylating ligase and MdmX levels cannot be rescued by CA-Src (bottom). **(B)** Endogenous levels of MdmX and Mdm2 increase with EGF treatment and this increase is attenuated with PP1 treatment. **(C)** Similar transfection as in A, but with various MdmX mutants showing that the CA-Src/Mdm2 mediated increase in MdmX levels is independent of these mutations. Asterisks indicate non-specific bands. All panels: number underneath the lanes represent the ratio of the intensity of the MdmX band to the intensity of the actin band as determined by ImageJ.

Mdm2 acts as a neddylating ligase in response to Src phosphorylation [23], but the effect on MdmX is unknown. When MdmX was transiently co-expressed with Mdm2 in H1299 cells, MdmX levels decreased dramatically compared to MdmX expression alone (Figure 1A top panel). This result was expected as down-regulation of MdmX in response to overexpression of Mdm2 has been reported in the literature [24, 25]. This affect was reversed with the addition of constitutively active Src (CA-Src). To show that these observations were dependent on the Src phosphorylation sites on Mdm2, we used the Src phosphorylation double mutant Y281/302F Mdm2 in the presence of CA-Src and MdmX. We found that CA-Src failed to rescue MdmX levels in the presence of the Mdm2 Y281/302F mutant (Figure 1A bottom panel). These data provide evidence that Src phosphorylation of Mdm2 prevents Mdm2-mediated destabilization of MdmX.

We next examined if we could see similar results in an endogenous system through pharmacological approaches. Serum starved MCF7 cells were treated with DMSO and EGF (epidermal growth factor), or PP1 (an inhibitor of Src) and EGF. Mdm2 and MdmX increased in the presence of EGF (Figure 1B). This increase in MdmX levels was attenuated in the presence of PP1. While PP1 blocks c-Src activity, Src abundance was not affected under these conditions (Figure 1B). These endogenous results show that MdmX levels are sensitive to the Src/Mdm2 pathway.

Compared to Mdm2 and p53, there is much less known about protein interactions that affect MdmX levels and function. When Y99 of MdmX is phosphorylated by c-Abl, interaction with p53 is prevented [26]. In addition, c-Abl drives Mdm2-MdmX complex formation, which leads to degradation of this complex [16]. Mutation of P369G disrupts binding to 14-3-3_**γ**_, which normally occurs upon irradiation exposure to UV to inhibit p53 ubiquitination [27]. We tested if a Y99F and a P369G mutant of MdmX were altered in response to Src signaling by transient expression in H1299 cells. Similar to the results shown in Figure 1A, the levels of MdmX, as well as each of the mutants, increased in the presence of CA-Src (Figure 1C). Overall, these results show that growth signaling results in increased protein levels of MdmX.

### Mdm2 and MdmX interact under growth conditions

**Figure 2:**
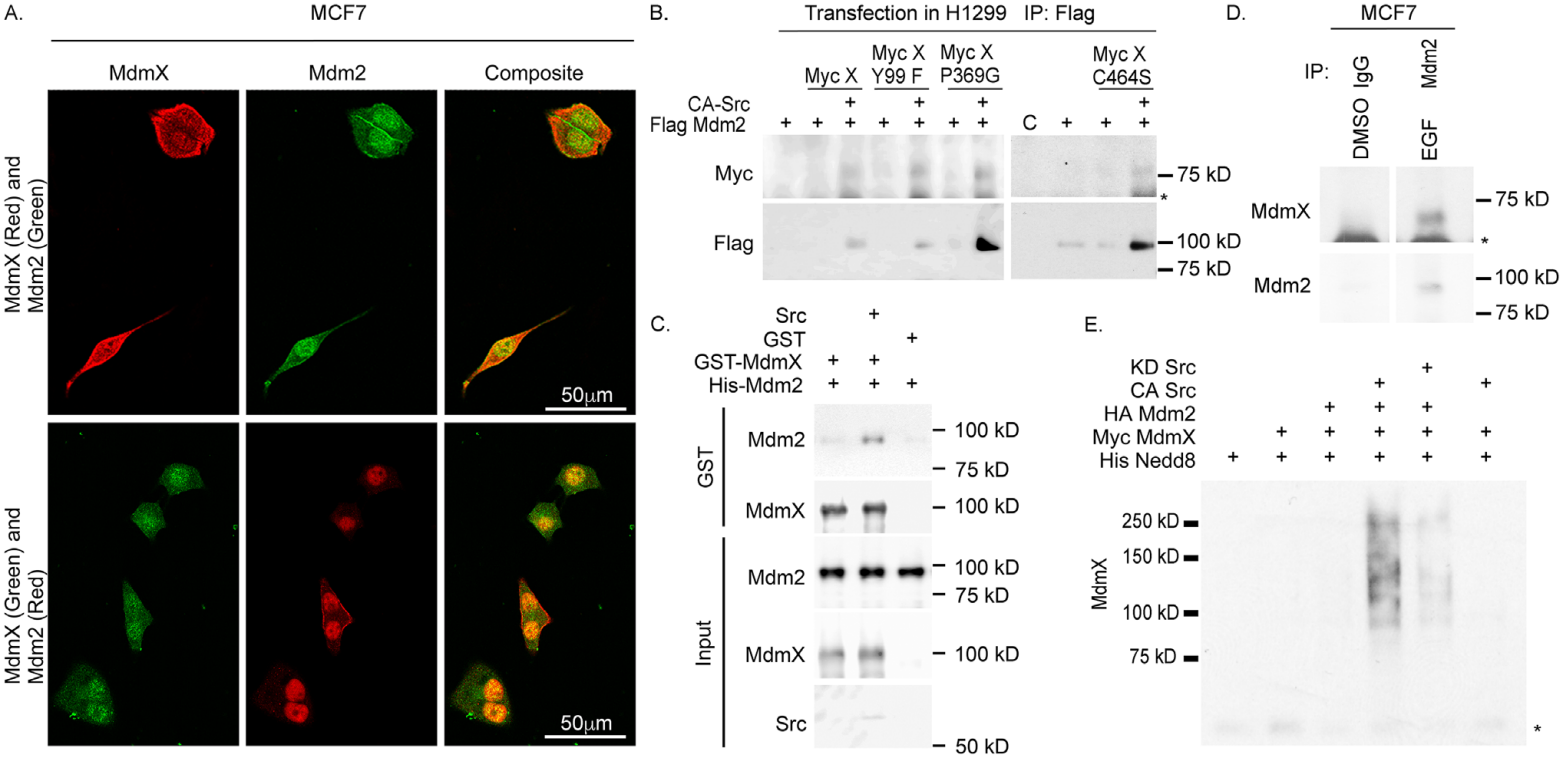
MdmX and Mdm2 interact under growth conditions **(A)** Confocal microscopy shows that MdmX and Mdm2 (red and green respectively in the top row or green and red respectively in the bottom row) are co-localized in the nucleus. **(B-D)** MdmX is in a complex with Mdm2 as determined from western blot analysis of immunoprecipitated extracts from transiently transfected H1299 cells (B), recombinant GST-Pulldowns (C), and immunoprecipitated MCF7 whole cell lysates (lanes spliced from the same gel after formatting) (D). **(E)** Western blot of a nickel pull-down of His-tagged Nedd8 showing that MdmX is neddylated in a CA-Src and Mdm2 dependent fashion. Asterisks indicate non-specific bands.

Since MdmX levels were dependent on Mdm2, we wanted to determine where MdmX and Mdm2 were localized in the cell under growth conditions (+EGF). Using immunofluorescence confocal microscopy we show that MdmX and Mdm2 were predominantly co-localized in the nucleus, but also in the cytoplasm of MCF7 cells (Figure 2A).

We then investigated if MdmX would co-purify with Mdm2. Indeed, in response to active Src, Mdm2 co-purified with MdmX when transiently expressed in H1299 cells (Figure 2B). In addition, every MdmX mutant tested was able to co-purify with Mdm2 in the presence of CA-Src (Figure 2B). We observed that the amount of Mdm2 immunoprecipitated in the presence of Src was increased in our experiments. To ensure that the increase in interaction between MdmX and Mdm2 was due to post-translational modifications and not due solely to increased expression levels, we used a cell free approach with bacterially produced recombinant proteins and found that the interaction between GST- Mdm2 and His-MdmX was increased in the presence of active Src (Figure 2C). This finding suggests that binding of Mdm2 with MdmX is not an artifact of increased protein levels and the increase in association was in response to phosphorylation.

To validate these findings, the endogenous interaction between MdmX and Mdm2 under growth conditions was investigated. MdmX co-purified with Mdm2 that was immunoprecipitated from EGF treated MCF7 cells (Figure 2D). Taken together, these results demonstrate that under growth conditions, when Mdm2 is phosphorylated by Src and acting as a neddylating enzyme, Mdm2 interacts with and increases MdmX.

The complex formation and increased MdmX abundance could be a result of neddylation by Mdm2. To test this, we transiently transfected H1299 cells with combinations of His-Nedd8, Myc-MdmX, HA-Mdm2 and CA-Src and performed a nickel pull-down assay. We found that MdmX was neddylated only in the presence of both Mdm2 and CA-Src (Figure 2E). This result also shows that in the presence of active Src, MdmX was unable to be neddylated in the absence of Mdm2 (Figure 2E). Reprobing the membrane showed that neddylation of Mdm2 also depends on Src (Supplementary Figure 1). Taken together, our data demonstrates that Mdm2 and MdmX form a neddylating complex that leads to neddylation of MdmX.

### MdmX enhances neddylation of p53

**Figure 3:**
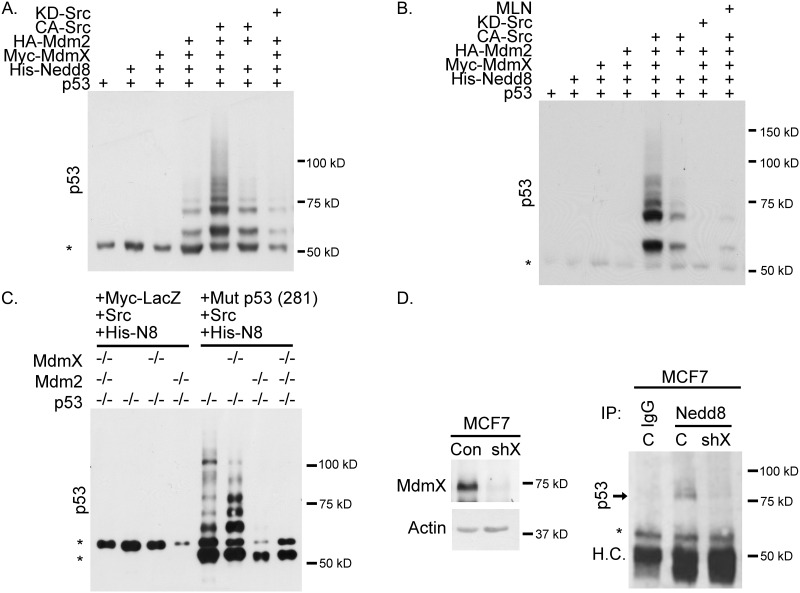
Wildtype and mutant p53 is neddylated in a MdmX-dependent manner as determined by western blot analysis **(A)** Nickel pull-down of His-Nedd8 showing that neddylation of p53 is dependent on MdmX and CA-Src. **(B)** Same Nickel pull-down as in A, but with the addition of MLN4924. MLN4924 decreases neddylation of p53. **(C)** Transient transfection of MEFs with CA-Src. His-Nedd8 and either Myc-LacZ or Mutp53. The presence of MdmX changes the banding pattern of neddylated p53. **(D)** MdmX was silenced in shMdmX MCF7 cells (left panel). Neddylated p53 was immunoprecipitated in the presence of MdmX, but not when MdmX was knocked down (right panel) (H.C.= Heavy Chain). All Panels: Asterisks indicate non-specific bands.

Considering that Mdm2 and MdmX form an active neddylating complex, we next examined if this complex was able to neddylate p53. A nickel pull-down assay showed that neddylation of p53 was more robust in the presence CA-Src and MdmX (Figure 3A and 3B). In the absence of exogenous MdmX, p53 neddylation was noticeably decreased. As predicted, transfection of kinase dead Src (KD-Src) resulted in less p53 neddylation compared to no Src overexpression (Figure 3A and 3B). To provide additional support for the requirement of an Mdm2-MdmX neddylation complex, we repeated the nickel pull-down assay and included MLN4924. Treatment with MLN4924 diminished neddylation of p53 despite expression of Mdm2, MdmX, and CA-Src (Figure 3B). Next, we transiently transfected mutant p53 D281G (to prevent lethality caused by p53 expression), CA-Src, and His-Nedd8 into p53-/-, p53 -/- MdmX -/-, p53-/- Mdm2-/-, and p53-/- MdmX-/- Mdm2 -/- MEFs. A nickel pull-down was then conducted, and while the presence of Mdm2 alone was sufficient for the faster migrating forms of neddylated p53, MdmX was necessary for the slower migrating forms of neddylated p53 (Figure 3C) in a murine system.

To further our analysis, we immunoprecipitated endogenous Nedd8 from shControl and shMdmX MCF7 cells and blotted for p53. We found that neddylated p53 was observed in shControl cells, but not in the shMdmX cells (Figure 3D; total p53 levels shown in Supplementary Figure 2). This endogenous data provides insight into signaling and neddylation of p53 under physiological conditions. Collectively, our data supports a key role for MdmX in enhancing Mdm2-mediated neddylation of p53.

### MLN4924 treatment results in decreased levels of MdmX

**Figure 4:**
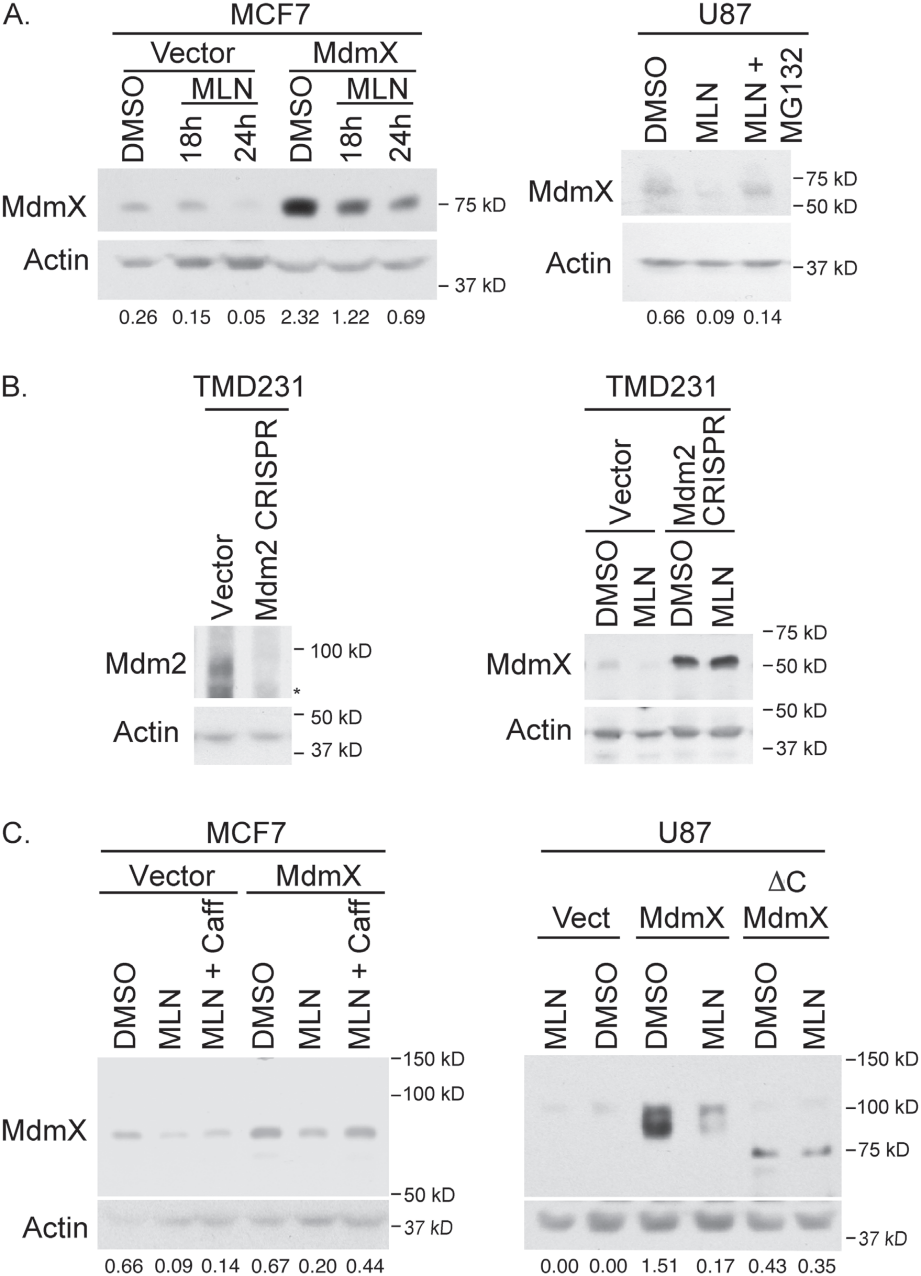
Westerns showing that MLN4924 decreases MdmX levels and this decrease depends on the proteasome, Mdm2, the C-terminus of MdmX and ATM activation **(A)** MLN4924 treatment decreases MdmX levels in both MCF7 (left panel) and U87 (right panel). Concurrent treatment with 20 μM MG132 (Velcade) rescues MdmX levels. **(B)** CRISPRed TMD231 cells that lack full length Mdm2 (left panel) do not have decreased MdmX in response to MLN4924 (right panel). **(C)** Co-treatment with 2 mM caffeine (ATM inhibitor) prevents the MLN4924-mediated decrease in MdmX levels. Therefore, intact signaling through the ATM pathway is required. Also, MdmX lacking the C-terminus is unresponsive to MLN4924. Panels A and C: number underneath the lanes represent the ratio of the intensity of the MdmX band to the intensity of the actin band as determined by ImageJ.

Since MdmX is neddylated (Figure 2E) and is involved in neddylating p53 (Figure 3), we wanted to investigate the effect of inhibiting neddylation with MLN4924, a small molecule inhibitor of Nedd8-activating enzyme. As expected, incubation of MCF7 and U87 cells with 0.3 μm MLN4924 resulted in decreased protein levels of MdmX (Figure 4A). Furthermore, this decrease was abrogated with treatment of 20 μM MG132, which suggests that MLN4924 results in proteosomal degradation of MdmX.

Since Mdm2 can ubiquitinate MdmX and target it for degradation through the proteosome, we examined whether Mdm2 was necessary for this MLN4924-mediated decrease of MdmX. Considering that genetically removing Mdm2 from cells that are wild type for p53 is lethal, we knocked out Mdm2 using CRISPR/Cas9 targeted to Exon 12 of Mdm2 in TMD231 cells [a derivative of MDA231 (mutant p53 R280K)]. After confirmation of Mdm2 knockout (Figure 4B left panel), cells were treated with MLN4924. As expected, a decrease in MdmX levels was detected in the vector control cells, which was not evident in the Mdm2 knock-out cells (Figure 4B right panel). This result verifies that MdmX degradation in response to MLN4924 is dependent on Mdm2.

To further explore this mechanism of Mdm2-mediated MdmX destabilization in response to MLN4924, we generated several MCF7 and U87 cell lines by viral transduction: vector control, wildtype MdmX, or MdmX with a C-terminal truncation that lacks a RING domain (ΔC MdmX) and is unable to interact with Mdm2 [14]. Figure 4C shows that, similar to Figure 4A, MLN4924 treatment decreased wildtype MdmX levels. However, this drug had no effect on ΔC MdmX, suggesting that binding with Mdm2 (via the RING domain) is necessary for MLN4924 mediated decrease of MdmX.

The Mdm2-mediated decrease of MdmX could be due solely to the inhibition of neddylation by MLN4924. Alternatively, we postulated that MLN4924 could be converting Mdm2 to be a ubiquitinating enzyme as MLN4924 was previously reported to activate ATM [28]. ATM has been shown to phosphorylate Mdm2 [3, 4, 29, 30] and MdmX [31] resulting in MdmX degradation after genotoxic stress. To determine if ATM was involved in MLN4924-mediated degradation of MdmX, we treated MCF7 cells with a combination of MLN4924 and caffeine (an ATM inhibitor). Inhibiting ATM rescued the MLN4924-mediated decreased in wildtype MdmX levels (Figure 4C). This result was validated with a more specific ATM inhibitor (KU-55933) (Supplementary Figure 3). Taken together, these results suggest that MLN4924 activates ATM, which then results in Mdm2-mediated degradation of MdmX through the ring domain of MdmX.

### p53 is phosphorylated, stabilized and activated by the MLN4924-ATM-Mdm2-MdmX pathway

**Figure 5:**
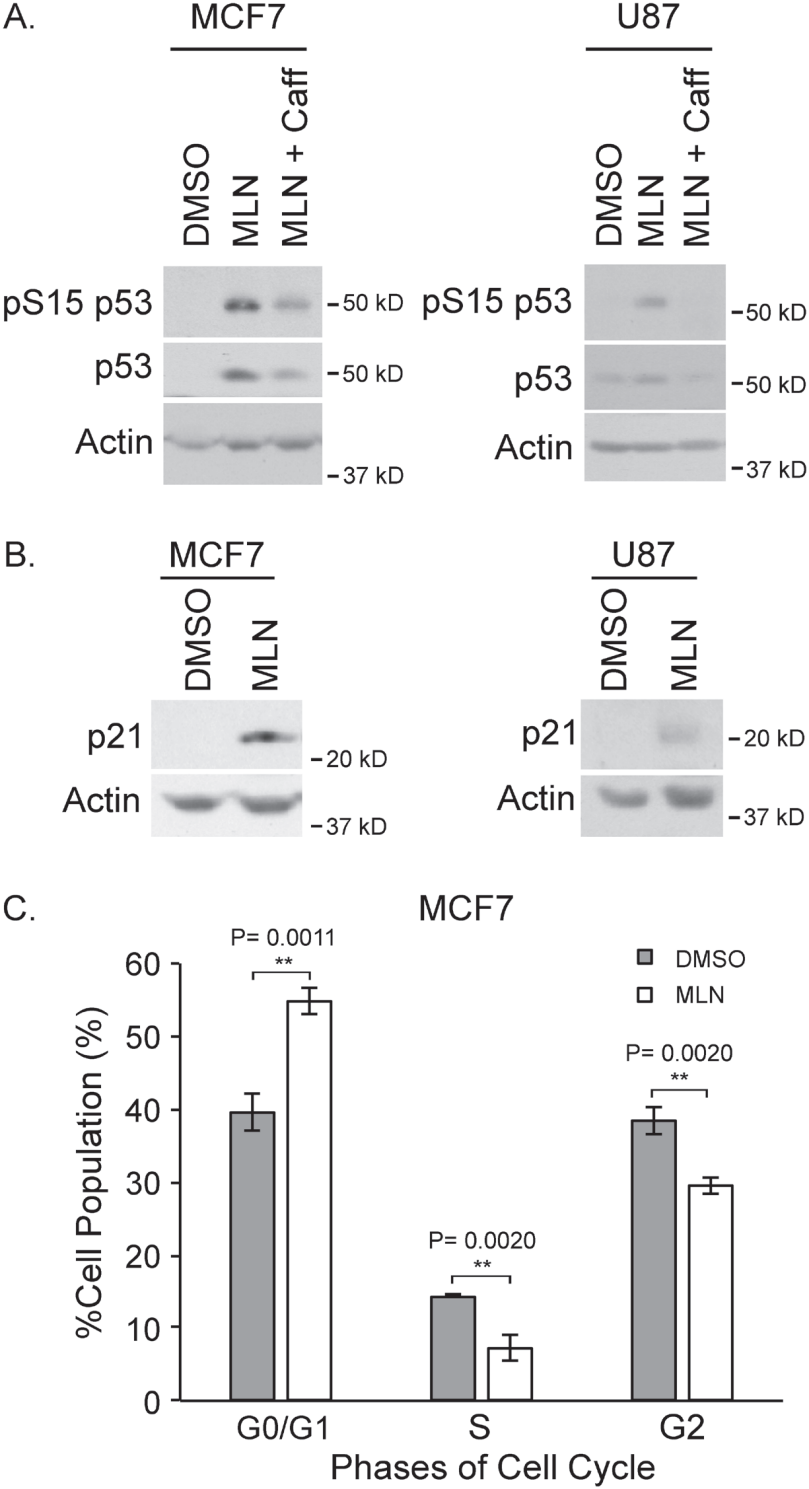
MLN4924 results in p53 activation and cell cycle arrest **(A)** Western blots of MCF7 (left) and U87 (right) extracts showing that S15 of p53 is phosphorylated in response to MLN4924 treatment but not with the addition of caffeine. **(B)** Western analysis of extracts from MCF7 (left) and U87 (right) cells showing increased levels of p21 in response to MLN4924. **(C)** Flow cytometry of MCF7 cells showed MLN4924-treated cells were arrested in G1.

As p53 is a substrate for ATM, we wanted to confirm that ATM was activated by MLN4924. Upon MLN4924 treatment, pS15 of p53 (an established ATM phosphorylation site) was detected, thus indicating ATM activation (Figure 5A). This phosphorylation was attenuated with the addition of caffeine, supporting that ATM was the responsible kinase. In addition, the amount of total p53 increased with MLN4924 treatment and this result was mitigated with the combination of MLN4924 and caffeine (Figure 5A).

Previously, we have shown that increased p53 levels do not necessarily result in increased activity. To assess p53 activity, we investigated the effect of MLN4924 treatment on p21 and found that p21 was also increased with MLN4924 treatment (Figure 5B). This result suggests that p53 is transcriptionally active upon MLN4924 treatment.

Lastly, we conducted fluorescence-activated cell sorting to determine if there were any aberrations in the cell cycle. Treatment of actively growing MCF7 cells with MLN4924 for 18 h resulted in a higher proportion of cells in the G1 phase and less cells in the G2 phase of the cell cycle (Figure 5C). Disruption of cell cycle upon MLN4924 is consistent with previous reports in Urothelial carcinoma [32], Ewing Sarcoma [33], Gastric cancer [28], melanoma [34] and breast cancer [35].

## DISCUSSION

It is well established that Mdm2 and MdmX can form heterodimers via their RING finger domains [9, 13–15]. However, how the complex is formed and the possible function(s) of this heterodimer under growth signaling has not been explored. We show that under growth conditions, MdmX is increased, the MdmX-Mdm2 heterodimer does form, and MdmX is neddylated. This is the first evidence that growth conditions regulate an increase of an oncoprotein complex.

Furthermore, since MdmX levels of all mutants were increased under conditions of growth, signaling through those pathways dependent on the wild-type residues are unlikely to be involved. Although the C464S mutant was tested (Figure 2B), it has been shown that MdmX overexpression can compensate for ring domain mutants of Mdm2 [9] and one can surmise that the reverse is also true. Since Mdm2 is stabilized when Src is active [23], it is possible that it is able to rescue the C464S mutant of MdmX.

There has been considerable interest in the development of therapeutics that modulate the regulation of kinase pathways and limited development on those that impact other post-translational modifications. One such small molecule, MLN4924, is a specific inhibitor of the NEDD8-activating enzyme in Phase 2 clinical trials. However, its effect on the regulation of p53 has not been fully explored. There have been reports that MLN4924 can elicit DNA stress type responses [35–38] in addition to preventing neddylation.

The majority of studies on the Mdm2-MdmX interaction have been conducted under conditions of DNA damage. We have shown that c-Abl phosphorylation of Mdm2 results in Mdm2-MdmX complex formation and ubiquitination of MdmX [16]. Other studies have shown that ATM activation after DNA damage results in ATM-mediated phosphorylation of Mdm2, which blocks nuclear export of p53 [30] and MdmX, which results in rapid destabilization of MdmX. In addition, ATM phosphorylation prevents poly-ubiquitination of p53 by inhibiting homodimerization of Mdm2 [4]. DNA damage has also been reported to result in checkpoint kinase (Chk) [27, 31, 39, 40] and ribosomal stress pathway [3, 41–44] activation, similarly resulting in MdmX degradation.

Another component that could be contributing to MdmX degradation under DNA damaging conditions is the activation of NEDP1. This isopeptidase is induced by chemotherapy and can de-neddylate Mdm2 [45]. It is conceivable that the DNA damage response that is initiated by MLN4924 treatment is activating NEDP1, which then de-neddylates Mdm2 and/or MdmX resulting in loss of MdmX. Our study illustrates that MdmX stability is sensitive to MLN4924.

The seminal finding of this study is the contribution of MdmX to the neddylation of p53. Initial work suggested the involvement of MdmX as part of a neddylating complex and reported that while co-expression of MdmX did not significantly affect Mdm2-mediated neddylation of p53, it was sufficient to rescue Mdm2 mutants that had impaired neddylation activity in transient transfections [9]. However, the mechanism by, and conditions under, which Mdm2 switches from ubiquitinating to neddylating activity was not yet known. In the current study, we show by transient overexpression in H1299 cells that the maximum amount of neddylated p53 is absolutely dependent on MdmX. Furthermore, if CA-Src is replaced with KD-Src or the cells are treated with MLN4924, this neddylation is diminished. In MEFs, the neddylation pattern is significantly different when MdmX is present compared to when it is absent. Importantly, MdmX contributes to neddylation endogenously as immunoprecipitation of Nedd8 from shMdmX MCF7 cells, followed by western blotting shows much less or almost no neddylated p53 compared to the control. Thus, corroborated by multiple experimental models and approaches, we show that MdmX greatly enhances the neddylation capacity of Mdm2.

**Figure 6:**
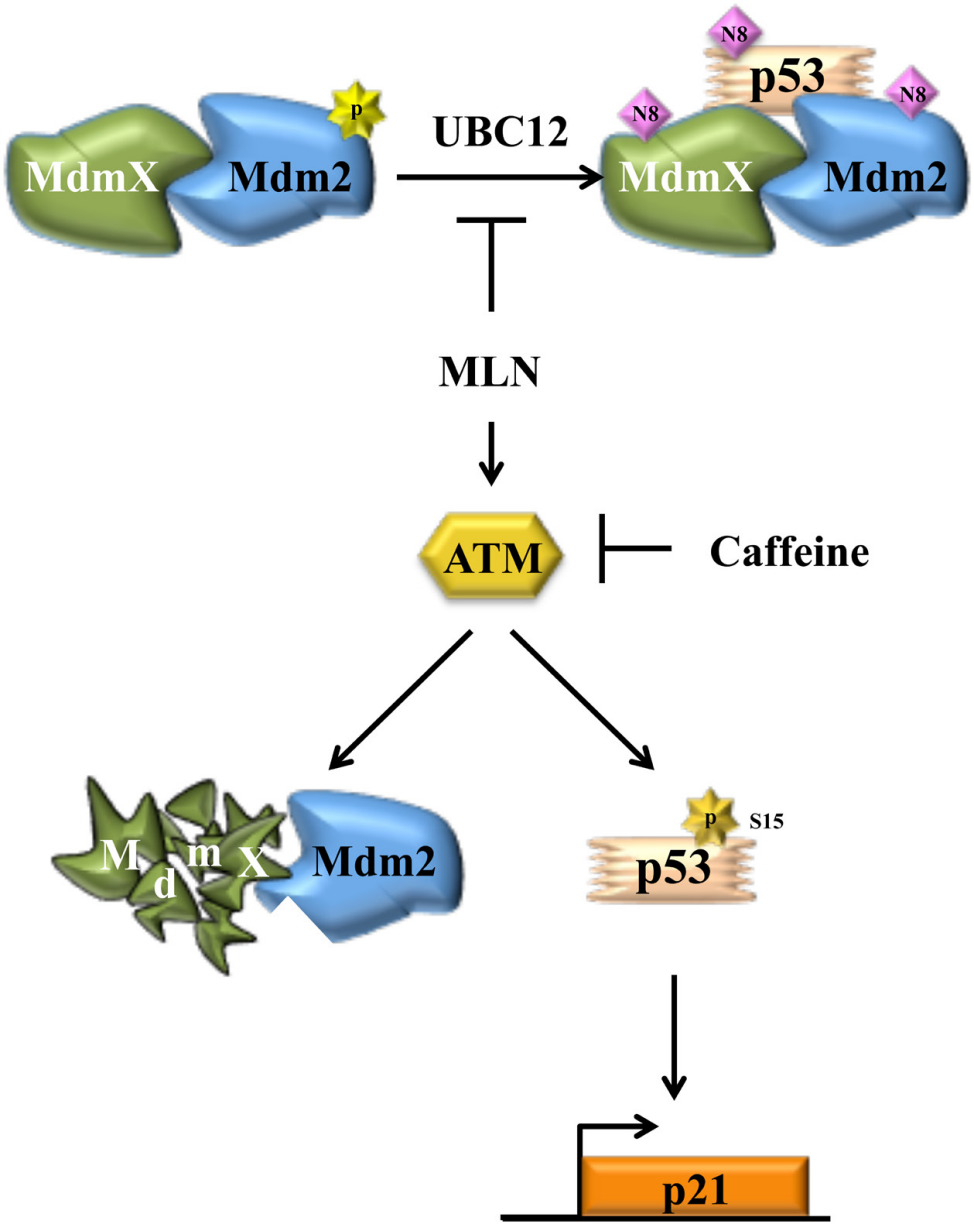
Illustration of how MdmX is integrated into growth factor-mediated neddylation Under growth conditions, Mdm2 and MdmX form a complex that is necessary to neddylate MdmX and p53. This function is inhibited by MLN4924 which also activates ATM. ATM activation results in conversion of Mdm2 to a ubiquitinating complex, which leads to MdmX destabilization, but not p53 degradation.

Taken together, our results show that during growth conditions when Src is active, Mdm2 and MdmX are in a complex together to neddylate p53 and MdmX. Inhibition of this activity with MLN4924 results in ATM activation, MdmX degradation, and increased p21 (Figure 6). This study highlights the complexity of signaling pathways that affect Mdm2-MdmX enzymatic activity within the cell and emphasizes the need to carefully consider this intricacy in future studies or when targeting this pathway for therapeutic intervention.

## MATERIALS AND METHODS

### Cell culture and treatments

The shMdmX, MdmX, and ΔC MdmX constructs were obtained [46]. Lentiviral CRISPR to Mdm2 was created with forward and reverse Mdm2 CRISPR primers (CACCGTTGGGCCCTTCGTGAGAAT and AAACATTCTCACGAAGGGCCCAAC respectively) to exon 12 cloned into the Lenticrispr vector (pXPR_001 obtained from Clark Wells) as described [47].

Viral transductions were conducted as described [48]. H1299, U87, TMD231, MEF and MCF7 cells were incubated at 37°C in a humidified chamber with 5% CO_2_ in DMEM high glucose and 8-10% fetal bovine serum. Transient transfections were performed using 1:1 DNA to PEI (H1299). Equal amounts of DNA were transfected into cells except for neddylation assays (see figure legend). Unless indicated, cells were treated with 0.3 μm MLN4924 (Life Sensors), 2 mM Caffeine and/or 10 μM ATMi (KU-55933 (Millipore) for 18 h.

### Protein analysis

Cell pellets were lysed in Urea Lysis Buffer (20mM Tris pH6.8, 100 mM NaH_2_PO_4_, 6M Urea) on ice for 2 hours and sonicated before centrifugation. After quantitation, proteins were analyzed by western blotting. All antibodies are listed in Supplementary Table 1.

### Immunoprecipitation

Cells were lysed in 25 mM Tris-HCl, 150 mM NaCl, 1% IGEPAL, protease cocktail, 1 mM Na_3_VO_4_, 10 mM NaF, 1 μm Sodium Pyrophosphate (pH 8.0) and sonicated. Antibodies were preabsorbed, washed and then incubated with lysate at 4°C for 2 h. All antibodies are listed in Supplementary Table 1.

### Kinase and other *in vitro* reactions

Kinase reactions were performed at 30°C for 60 minutes in kinase buffer (60 mM HEPES, pH 7.4, 3 mM MgCl_2_, 3 mM MnCl_2_, 3 μm Na_3_VO_4_, 20 mM ATP) using 0.1 μg of Src (Millipore). Kinase reactions were incubated with glutathione-sepharose beads that were pre-washed in 0.1% DTT for 30 minutes. This incubation was conducted for 10 minutes in a 0.1% DTT buffer. Complexes that formed were spun down and washed four times in a high salt buffer (0.5 M KCl, 20 mM Tris, 0.1% IGEPAL, pH 8.0). The bound proteins were eluted in SDS-Loading Buffer and western blot analysis was performed.

### Nickel pull down

H1299 cells were transfected with His-nedd8 and other plasmids using PEI (1 DNA:1 PEI ratio). Twenty-four hours after transfection, cells were lysed in 500 μl of 6 M guanidinium–HCl, 0.1 M Na_2_HPO_4_/NaH_2_PO_4_, 0.01 M Tris–HCl pH 8.0 plus 5 mM imidazole and 10 mM ß-mercaptoethanol. After centrifugation, the lysates were mixed with 30 μl of Ni^2+^-NTA-agarose beads (Qiagen) prewashed with lysis buffer and incubated for 2 h at room temperature. The beads were successively washed in each of the following: 6 M guanidinium–HCl, 0.1 M Na_2_HPO_4_/NaH_2_PO_4_, 0.01 M Tris–HCl pH 8.0 plus 10 mM ß-mercaptoethanol; 8 M urea, 0.1 M Na_2_HPO_4_/NaH_2_PO_4_, 0.01 M Tris–HCl pH 8.0, 10 mM ß-mercaptoethanol; 8 M urea, 0.1 M Na_2_HPO_4_/NaH_2_PO_4_, 0.01 M Tris–HCl pH 6.3, 10 mM ß-mercaptoethanol plus 0.2% Triton X-100; 8 M urea, 0.1 M Na_2_HPO_4_/NaH_2_PO_4_, 0.01 M Tris–HCl pH 6.3, 10 mM ß-mercaptoethanol plus 0.1% Triton X-100. After the last wash the beads were eluted with 200 mM imidazole in 5% SDS, 0.15 M Tris–HCl pH 6.7, 30% glycerol, 0.72 M ß-mercaptoethanol. Eluates were subjected to sodium dodecyl sulfate polyacrylamide gel electrophoresis (SDS–PAGE) and western blotting.

### Confocal analysis

MCF7 cells growing on coverslips were serum starved for 72 hours and then treated for EGF for 8 hours. Cells were washed in PBS and then fixed for one hour at room temperature in 4% paraformaldehyde. After 3 washes in PBS, cells were permeabilized in 0.1% Triton-X 100 for 20 minutes. and washed 3X in PBS. After blocking in 2% BSA, coverslips were incubated with MdmX and/or Mdm2 antibodies (Red Mdm2 – N20, Green Mdm2 – SMP14, Red MdmX – Bethyl, Green MdmX – 8C6) overnight at 4° C and washed 3X in PBS. Coverslips were then incubated with AlexaFluor 488 and 637 linked to anti-mouse and anti-rabbit secondary antibodies (respectively) for 1 hour, washed 3X in PBS, mounted on slides with Prolong Diamond Antifade Mountant with DAPI and visualized with a Leica SP8 MP confocal microscope at room temperature. Images were obtained using HC PL APO 40x/1.3 oil CS2 objective and the Leica Application Suite Advanced Fluorescence Software provided by the Indiana Center for Biological Microscopy core.

### Cell cycle analysis

MCF-7 cells (3×10^5^) were plated onto 6-well plates in triplicates and serum starved for 24 h. Cells were released from starvation with DMEM complete and treated with DMSO or MLN (0.6 μM) for 18 h, cells were washed with 1X PBS, harvested with trypsin, and spun at 300g x 5 minutes. FACS analysis was performed on the Muse (Millipore) with the Muse Cell Cycle Kit, according to manufacturers instructions (5,000 events). Significance was calculated using Two-tailed Student’s T-Test.

## SUPPLEMENTARY MATERIALS FIGURES AND TABLE



## Abbreviations

Mdm2: Mouse double minute 2
MEFs: mouse embryonic fibroblasts
ATM: Ataxia Telangiectasia Mutated
RING: Really Interesting New Gene
EGF: epidermal growth factor
c-Abl: Abelson murine leukemia viral oncogene homolog 1
DMSO: Dimethyl sulfoxide
CRISPR: Clustered regularly interspaced short palindromic repeats
chk: checkpoint kinase
Nedd8: Neural Precursor Cell Expressed, Developmentally Down-Regulated 8
NEDP1: NEDD8-Specific Protease 1
CA: constitutively active
KD: kinase dead
C: control
WB: western blot
sh: short hairpin
HA: Human influenza hemagglutinin aa 98-106
Caffeine: Caff
