# Supplementary material for: The fate of murine double minute X (MdmX) is dictated by distinct signaling pathways through murine double minute 2 (Mdm2)
Source: Oncotarget. 2017 Nov 6;8(61):104455–66. doi: 10.18632/oncotarget.22320 (PMC5732819; doi:10.18632/oncotarget.22320)
Supplement: Supplementary file 1 [file oncotarget-08-104455-s001.pdf]

The fate of murine double minute X (MdmX) is dictated by distinct signaling pathways through murine double minute 2 (Mdm2)

SUPPLEMENTARY MATERIALS

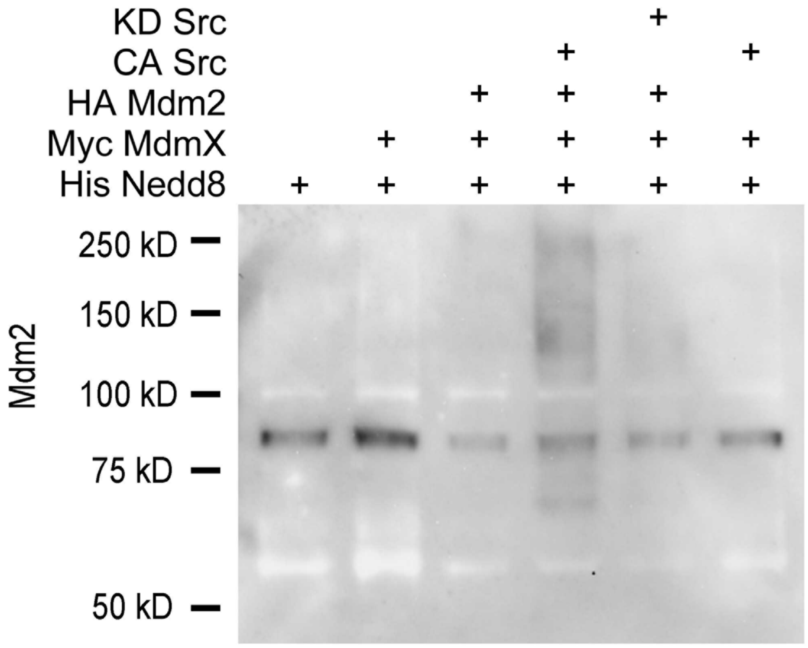

Supplementary Figure 1: Mdm2 is neddylated with MdmX and CA-Src. The membrane from Figure 2E was reprobed with Mdm2 (H221 and N-20 Santa Cruz Biotech).

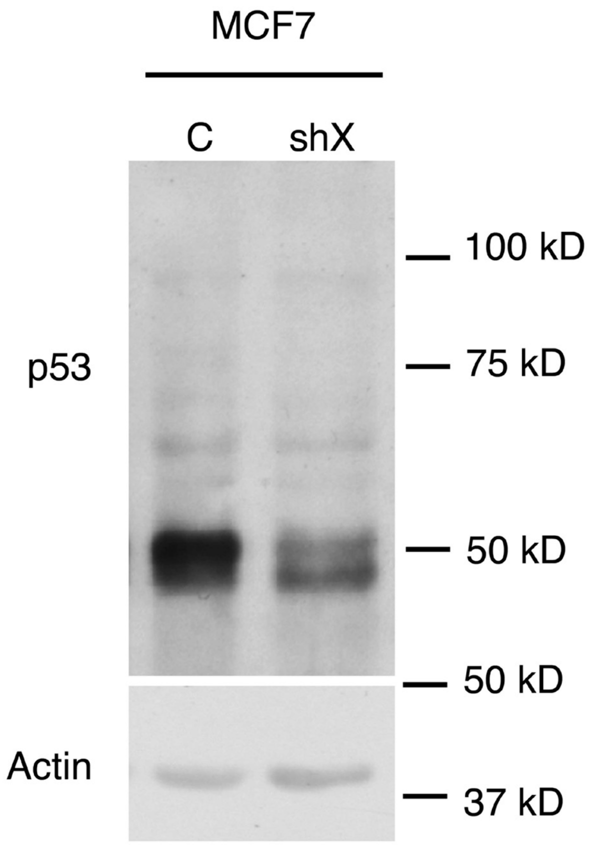

Supplementary Figure 2: Western blot analysis of p53 levels in whole cell extracts used in Figure 3D. As expected, specific neddylated forms of p53 are less abundant without MdmX to contribute to Mdm2-mediated neddylation and p53 stabilization under growth conditions.

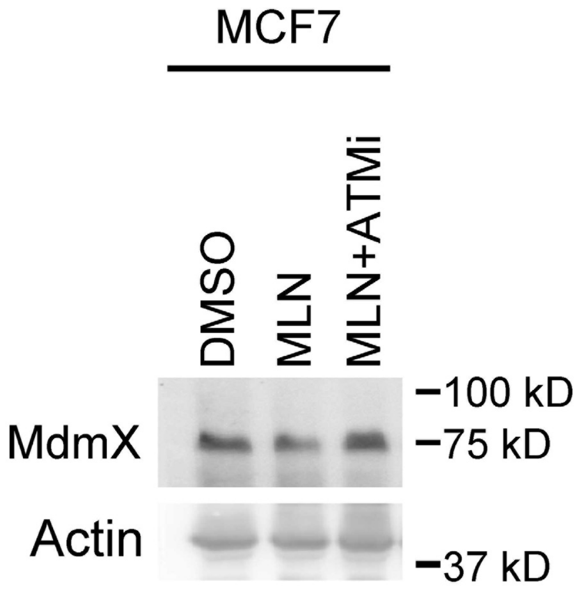

Supplementary Figure 3: Western blot analysis demonstrating that treatment with ATM Kinase Inhibitor attenuates MLN-mediated decrease of MdmX. MCF7 cells were treated with 0.3 mM MLN with or without 10 mM KU-55933 (ATMi) for 16 hours. These results are similar to those observed with caffeine treatment.

Supplementary Table 1: Antibody list

Antibody	clone/epitope	Secondary	Company	Catalogue number	Lot number
Actin	AC-15	Mouse	Sigma	A1978	065M-4837
HA	12CA5	Mouse	Roche	11583816001	10952100
Flag	FG4R	Mouse	Thermo	MA1-91878	PG202689,
GST	56C1	Mouse	Santa Cruz	sc-80998	H0613
Mdm2	SMP14	Mouse	Santa Cruz	sc-965	B2516
	IF2	Mouse	Cal. Biochem	OP-46	2822778, D00158850
	N-20	Rabbit	Santa Cruz	sc-813	J0412
MdmX		Mouse	Bethyl	A300-287A	A300-287A-1
	8C6	Mouse	Millipore	04-1555	2716342, 2780486
Myc	9E10	Mouse	Santa Cruz	sc-40	E0615
Nedd8	Alexis	Rabbit	Alexis Biochemicals, Enzo Life Sciences	ALX-210-194-R200	
p21	C-19	Rabbit	Santa Cruz	sc-397	B0508, C1014
p53	pSer15	Rabbit	Cell Signaling	9284	8
p53	pSer15	Mouse	Cell Signaling	9286	3
p53	DO1	Mouse	Santa Cruz	sc-126	K2216, I2515
c-Src	B-12	Mouse	Santa Cruz	sc-8056	B0212

The antibodies, clone numbers or epitopes, corresponding secondary antibody, company, catalogue number, and lot number are described.
